# Room temperature near unity spin polarization in 2D Van der Waals heterostructures

**DOI:** 10.1038/s41467-020-18307-w

**Published:** 2020-09-07

**Authors:** Danliang Zhang, Ying Liu, Mai He, Ao Zhang, Shula Chen, Qingjun Tong, Lanyu Huang, Zhiyuan Zhou, Weihao Zheng, Mingxing Chen, Kai Braun, Alfred J. Meixner, Xiao Wang, Anlian Pan

**Affiliations:** 1 Key Laboratory for Micro-Nano Physics and Technology of Hunan Province, School of Physics and Electronics, Hunan University, Changsha, 410082 China; 2 Key Laboratory for Micro-Nano Physics and Technology of Hunan Province, College of Materials Science and Engineering, Hunan University, Changsha, 410082 China; 3 Key Laboratory for Matter Microstructure and Function of Hunan Province, School of Physics and Electronics, Hunan Normal University, Changsha, 410081 China; 4 Institute of Physical and Theoretical Chemistry and LISA+, University of Tübingen, Auf der Morgenstelle 18, 72076 Tübingen, Germany

**Keywords:** Nanoscience and technology, Optics and photonics

## Abstract

The generation and manipulation of spin polarization at room temperature are essential for 2D van der Waals (vdW) materials-based spin-photonic and spintronic applications. However, most of the high degree polarization is achieved at cryogenic temperatures, where the spin-valley polarization lifetime is increased. Here, we report on room temperature high-spin polarization in 2D layers by reducing its carrier lifetime via the construction of vdW heterostructures. A near unity degree of polarization is observed in PbI_2_ layers with the formation of type-I and type-II band aligned vdW heterostructures with monolayer WS_2_ and WSe_2_. We demonstrate that the spin polarization is related to the carrier lifetime and can be manipulated by the layer thickness, temperature, and excitation wavelength. We further elucidate the carrier dynamics and measure the polarization lifetime in these heterostructures. Our work provides a promising approach to achieve room temperature high-spin polarizations, which contribute to spin-photonics applications.

## Abstract (web summary)

In two-dimensional semiconductors, light can generate spin polarisations, however this effect is typically limited to low temperatures. By combining Lead Iodide (PbI_2_) with transition metal dichalcogenides (TMDCs), the authors demonstrate room temperature light induced near-unity spin polarisations.

## Introduction

Van der Waals (vdW) heterostructures formed by different two-dimensional (2D) materials are emerging as an excellent platform for both fundamental research and technical applications^1–3^, due to the unique photonic, optoelectronic properties of the individual materials, and the combination their advantages in heterostructures^4–7^. The band alignment at the interface of the vdW heterostructures can modulate the interfacial carrier behaviors, which is essential for device applications^8^. Up to now, vdW heterostructure-based logic transistors^9^, modulators^10^, light-emitting diodes (LEDs)^11^, fast photodetectors^12^, and photovoltaic cells^13^ have been realized, which make them important building blocks for photonic and optoelectronic devices and integrated circuits.

With the development of quantum information science, the 2D materials-based spin-photonic devices are highly desirable. The generation and control of spin polarization is at the heart of this field. One recent progress along this direction is the light-induced spin and valley polarization in transition metal dichalcogenides (TMDCs)^14–17^ and their heterostructures^18–22^. However, most of these studies were performed at low temperature with high-quality samples, so as to reduce the intervalley scattering and increase the spin-valley polarization lifetime^23,24^. It is therefore greatly desirable to find new materials and/or mechanism to obtain a high degree of spin polarization at room temperature, which is essential for practical spintronic applications.

Compared to typical TMDCs such as WS_2_ and WSe_2_, layered lead iodine (PbI_2_) has a wider bandgap and higher light absorption coefficient, showing potential optoelectronic applications^25–28^, such as photodetectors^29–32^, X-ray or γ-ray detection^33,34^, and optically pumped lasers^35^. PbI_2_ has a hexagonal crystal pattern composed of covalently bonded repeating sequences of I-Pb-I atomic layers, with weak vdW interaction between the two layers^25,26^. The bandgap can be tuned from a direct gap of 2.28 eV to an indirect-gap of 2.63 eV when reducing its thickness or a fine-tuning by applying strain^25,26^. Due to the vdW nature, layered PbI_2_ can easily form heterostructures with other TMDCs materials^36^, exhibiting versatile band alignment^37,38^. Furthermore, since PbI_2_ can be used as a precursor of lead halide perovskite, the conversion from PbI_2_/TMDCs to perovskite/TMDCs heterostructures have been realized^39,40^, which further extends the applications of PbI_2_. The formation of high-quality heterostructures provides the opportunity to manipulate the carrier dynamics in the vertical direction and affects the carrier lifetime^41^.

Here, we report on room temperature high-spin polarization in PbI_2_ layers via the construction of heterostructures with monolayer TMDCs. Due to the reduction of carrier lifetime, a near-unity degree of polarization at room temperature is observed from both layered PbI_2_/monolayer-WS_2_ type-I and PbI_2_/monolayer-WSe_2_ type-II band aligned heterostructures. The spin polarization related to the carrier lifetime can be manipulated by the material thickness, temperature, and excitation wavelength, providing versatile control means for further practical applications. In addition, we investigate the polarized carrier dynamics in heterostructures and reveal the polarization lifetime by time-resolved polarization experiments. This work not only provides a basis for controlling carrier dynamics and spin polarization in 2D vdW heterostructures but also offers a strategy to achieve a high degree of spin polarization at room temperature, promising for spin-photonics applications.

## Results

### PbI_2_/WS_2_ heterostructures and near-unity polarization

**Fig. 1:**
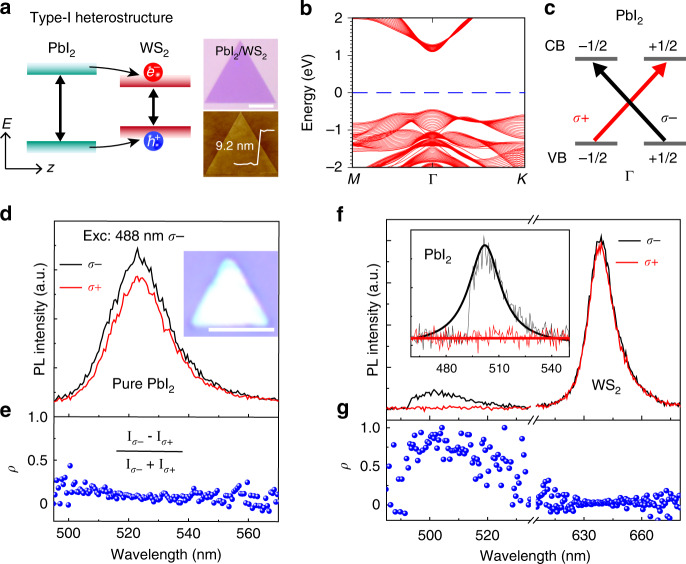
Spin polarization mechanism and spectrum of PbI_2_/WS_2_ heterostructures. **a** Schematic of the type-I heterostructure for the study of the carrier interlayer transportation. Both electrons and holes transfer from PbI_2_ to WS_2_. The images on the right are optical and AFM micrographs of a PbI_2_/WS_2_ heterostructure with a thickness of 9.2 nm. **b** Band structure for the PbI_2_ thin film with a thickness of 20 layers, which is spin degenerate. **c** Schematic illustration of the polarized optical transitions in PbI_2_ thin films. **d** Circularly polarized PL spectra from pure PbI_2_ at room temperature. **e** Corresponding degree of polarization *ρ* of pure PbI_2_ PL calculated from the PL spectra shown in figure (**d**). **f** Circularly polarized PL spectra of a representative PbI_2_/WS_2_ heterostructure at room temperature. **g** Corresponding degree of polarization *ρ* calculated from the PL spectra shown in figure (**f**). All length of the scale bar is 10 µm.

The heterostructures consisting of a bottom WS_2_ monolayer and top PbI_2_ layers with different thicknesses were prepared with a two-step physical vapor deposition (PVD) method. Typical heterostructures show a triangular shape with a uniform and smooth surface (Fig. 1a). Based on the calculated electronic structures from previous works^38,41^, the formed PbI_2_/WS_2_ heterostructures show a type-I band alignment, such that the photogenerated electrons and holes can be transferred from the PbI_2_ to the WS_2_ monolayer (Fig. 1a), which is also confirmed by our experimental results. Since circularly polarized photons carry angular momentum components, the absorption of a circularly polarized laser leads to the so-called optical spin injection to the materials^42^. Figure 1b shows the band structure for the PbI_2_ thin film with a thickness of 20 layers (details of density-functional theory calculations are given in Supplementary Note 1). Both the valence band maximum (VBM) and conduction band minimum (CBM) locate at the Г point and are spin degenerate because the thin film has the inversion symmetry and time-reversal symmetry. Figure 1c shows schematically the optical selection rules for PbI_2_ layers at Γ point. The right-handed (σ+) polarized photon carrying an angular monument of $+ \hbar$ causes the transition from the spin state −1/2 to +1/2, while left-handed (σ−) excitation referring to the transition from the spin state +1/2 to −1/2. The degree of photoluminescence (PL) circular polarization (*ρ*) can be defined as *ρ*_PL_ = (*Ι*_σ__−__/__σ__+_ − *Ι*_σ__+/__σ__−_)/(*Ι*_σ__+_ + *Ι*_σ__−_) under σ−/σ+ polarized excitation, where *Ι*_σ__+_ and *Ι*_σ__−_ denote the σ+ and σ− polarized PL intensities, respectively.

Experimentally observed polarized PL spectra (Fig. 1d) and the calculated *ρ* (Fig. 1e) from pure PbI_2_ layers (grown on SiO_2_/Si substrate, about 100 nm) show a small (*ρ* ≈ 0.1 for 488 nm excitation) degree of polarization and also an excitation wavelength dependence that further away from the resonance excitation leads to a much smaller value (Supplementary Fig. 5). Such a low polarization can be due to the spin relaxation during the carrier lifetime. Considering the carrier lifetime (*τ*_c_) and spin relaxation time (*τ*_s_), the measured degree of the polarization can be expressed as *P* = *P*_0_/(1 + *τ*_c_/*τ*_s_), where *P*_0_ is the polarization without spin relaxation^42^. For carrier lifetimes much longer than the spin relaxation (*τ*_c_ » *τ*_s_), the polarization will be small. In order to obtain a high polarization of *P*, one should either increase the spin relaxation time (*τ*_s_) or decrease the carrier lifetime (*τ*_c_). Although the former depends on the detailed material and experiment conditions and thus is hard to control, the latter can be engineered by stacking different 2D materials to form a vdW heterostructure. The mechanism behind is that in a vdW heterostructure the band alignment leads to interlayer charge transfer that would significantly reduce the carrier lifetime and thus increase the spin polarization. For pure WS_2_ monolayer, the circular polarization excitation creates the valley polarization and the degree of polarization is in general small under off-resonance excitation at room temperature (Supplementary Fig. 6)^43^.

The PbI_2_/WS_2_ heterostructures were excited with σ− excitation and the PL emission with σ+ and σ− polarizations were detected (Fig. 1f). For the PL signal from PbI_2_, we observe that the dominating emission is σ− polarized (black). Compared to the pure PbI_2_ layers, the degreed of polarization of PbI_2_ in heterostructures increases dramatically, almost reaching 100% at room temperature (Fig. 1g), consistent with our above mechanism. Explicitly, the carrier transfer process dramatically reduces the carrier lifetime of PbI_2_^41^, which leads to a small value of *τ*_c_/*τ*_s_ hence resulting in near-unity spin polarizations under resonance excitation. In contrast, the degree of polarization of the WS_2_ monolayer in heterostructures decreases, showing almost no polarization. Therefore, vdW heterostructures, where extra decay channels reduce the carrier lifetime largely, provide an excellent platform to achieve a high degree of polarization.

**Fig. 2:**
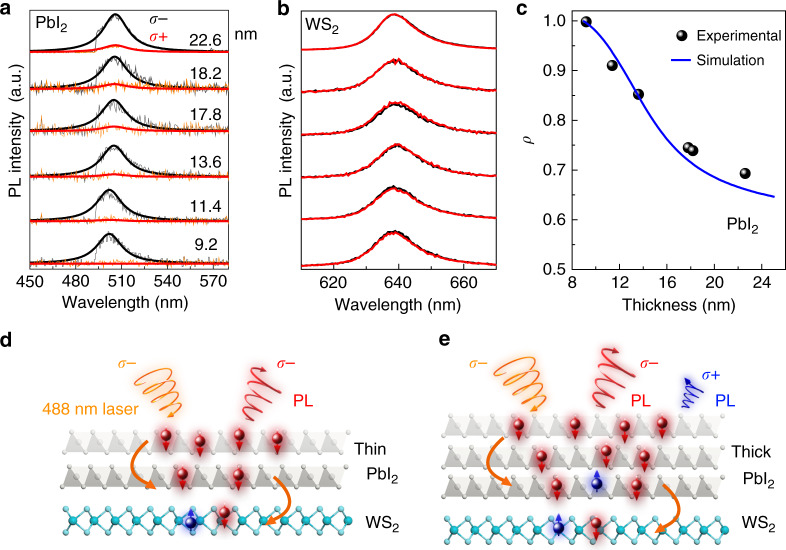
Thickness-dependent spin polarization and the underlying mechanism in PbI_2_/WS_2_ heterostructures. **a**, **b** Circularly polarized PL spectra of PbI_2_ and WS_2_ in PbI_2_/WS_2_ heterostructures with different thicknesses. **c** Degree of polarization *ρ* of PbI_2_ as a function of thickness of the heterostructures. The solid line represents the numerical simulation result. **d**, **e** Schematic illustrations of the resulting spin polarization in the thin and thick PbI_2_/WS_2_ heterostructures. The curled curves represent the incident light and the PL emissions. The red balls represent spin-down electrons, and the blue balls represent spin-up electrons.

To further verify the carrier lifetime enhanced the degree of polarization, we investigated how it depends on the thickness of the PbI_2_ layer while maintaining the bottom monolayer WS_2_ in the heterostructures. For pure PbI_2_ layers with different thicknesses, the PL shows similar decay curves in time-resolved PL (TRPL) spectra (Supplementary Fig. 7). In contrast, increasing the thickness of the PbI_2_ layer in heterostructures will increase its carrier lifetime, which would decrease the obtained polarization. Figure 2 displays the circularly polarized PL spectra from PbI_2_/WS_2_ heterostructures with the total thickness varying from 9.2 to 22.6 nm, with σ− excitation. For the PL signal from PbI_2_ (Fig. 2a), besides the overall dominating emission with σ− polarization (black), we observe that the weak emission signal from the σ+ polarization (red) increases with the increasing thickness of heterostructures. In contrast, the relative intensities of σ+ and σ− emission from the monolayer WS_2_ in the heterostructures are almost unchanged (Fig. 2b), showing the small degree of polarization as we discussed previously. With the spectra in Fig. 2a, we have calculated *ρ* for the PbI_2_ PL from different heterostructures and show that *ρ* decreases from 0.998 to 0.693 as the heterostructures thickness increases from 9.2 to 22.6 nm (Fig. 2c).

This phenomenon is related to the changing of carrier lifetime and can be understood by following the equation *P* (*X*) = *P*_0_/(1 + *τ*_eff(*X*)_/*τ*_*s*_), where *τ*_eff_ is the effective lifetime of the carriers, *X* is the thickness of the PbI_2_ layers (Supplementary Note 2). Based on the coupled rate equations of spin injected carriers, we performed simulations of the degree of polarization for PbI_2_ PL in heterostructures (solid line in Fig. 2c) as a function of heterostructure thickness. One can see that the simulations are in good agreement with our experimental data (solid spheres in Fig. 2c). We illustrate the phenomena in Fig. 2d, e for typical thin and thicker PbI_2_ in heterostructures that the spin-flip within the longer carrier lifetime leads to a smaller degree of polarization. We also measured excitation power dependent circularly polarized PL spectra, and reveal only a slight decrease of the *ρ* of PbI_2_ PL when the excitation power increases from 2 to 10 μW (Supplementary Fig. 8).

### Polarization dynamics

**Fig. 3:**
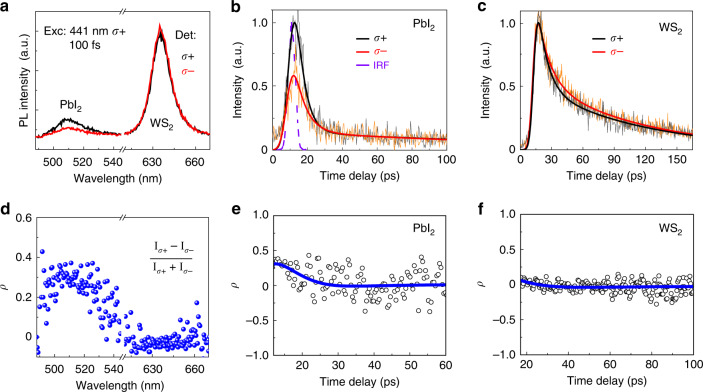
Spin polarization dynamics of PbI_2_/WS_2_ heterostructures. **a**–**c** Circularly polarized PL spectra (**a**) and TRPL data from PbI_2_ (**b**), and WS_2_ (**c**) of PbI_2_/WS_2_ heterostructures. **d**–**f** Corresponding degree of polarization *ρ* as a function of wavelength and time calculated from the PL and TRPL spectra. For excitation, a 441 nm *σ*+ polarized fs-pulsed laser beam was used.

To probe the polarization lifetime, we have excited a 13.6 nm thick PbI_2_/WS_2_ heterostructure with σ+ polarized 100-fs laser at 441 nm, and recorded the spectrally integrated and time-resolved σ+ and σ− PL emission, respectively. The integrated PL intensity (Fig. 3a) shows a degree of polarization of about 0.3 for PbI_2_, and almost zero degree of polarization for WS_2_ (Fig. 3c). Compared with the experiments under the excitation at 488 nm, we observed a reduced degree of polarization due to the off-resonance excitation, which is similar to the observations of many TMDCs atomic layers under different excitation photon energies^14,44^. Almost no polarization can be observed for the same heterostructures exited with 100 fs-pulsed laser at 400 nm, since they are even further off-resonance (Supplementary Fig. 9). In Fig. 3b, we show the σ+ (black) and σ− (red) PbI_2_ PL emission decay detected in the spectral range from 2.30 to 2.53 eV by a streak camera. The dashed line represents the instrument response function (IRF) obtained from pure laser pulses under the same experimental condition. After the deconvolution of the measured decay curves from the IRF and the fitting, we obtain the decay times for the σ+ and σ− polarized PbI_2_ PL emission, which are 15.7 and 27.5 ps, respectively. With these two time-resolved polarized PL decays, we can calculate the degree of polarization as a function of time, revealing the polarization lifetime of about 15 ps (Fig. 3e). For resonance excitation, the polarization lifetime is expected to be longer^45^, which could lead to a higher degree of polarization due to a smaller *τ*_c_/*τ*_s_. For the WS_2_ monolayer in the heterostructures, the σ+ and σ− polarized PL emissions show similar decay curves of about 49 ps (Fig. 3c), consistent with the integrated PL spectra, leading to the non-observable polarization lifetime (Fig. 3f). It should be noted that the dominating excited carriers account for the WS_2_ PL in heterostructures originate from the transferred carriers, not the optical valley initialization in pure WS_2_ monolayer.

### Temperature-dependent polarization

**Fig. 4:**
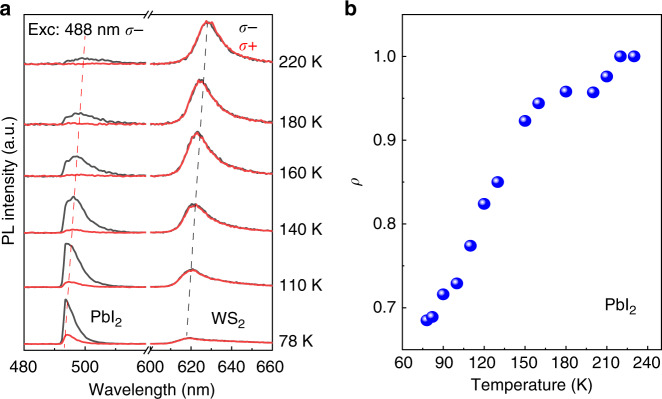
Temperature-dependent degree of polarization from PbI_2_/WS_2_ heterostructures. **a** Circularly polarized PL spectra of PbI_2_/WS_2_ heterostructure at different temperatures for excitation with a 488 nm *σ*− polarized CW laser beam. **b** Degree of polarization *ρ* of PL from PbI_2_ as a function of temperature, showing a decreasing tendency at lower temperatures.

Figure 4a shows the temperature-dependent circularly polarized PL spectra of PbI_2_/WS_2_ heterostructures. With decreasing temperature, the PL emission from PbI_2_ and WS_2_ blue shifts, as indicated by the dashed line. The PL intensity of PbI_2_ largely increases in comparison with that of WS_2_. This is because the interlayer charge transfer from PbI_2_ to WS_2_ is momentum mismatched. This process is only allowed with the assistance of phonons, which is suppressed greatly at low temperatures. As a result, the carrier lifetime in the PbI_2_ layer is increased with a decrease in temperature, similar to the effect of increasing layer thickness, which leads to a decrease in the degree of polarization *ρ*. Indeed, we found *ρ* of PbI_2_ PL in heterostructures decreases from 0.99 to 0.68 when the temperature decreases from 220 to 78 K (Fig. 4b). This temperature dependence is in contrast to the case for the pure TMDCs, where the degree of polarization in TMDCs monolayer normally increases at lower temperatures due to the reduced intervalley scattering. In control measurements with pure PbI_2_ layers, we find that the small *ρ* at room temperature slightly increases at low temperatures (Supplementary Fig. 11), which indicates that the observed temperature-dependent polarization in heterostructures does not originate from the intrinsic property of pure PbI_2_ but due to interlayer charge transfer process. Our results suggest that PbI_2_ in PbI_2_/WS_2_ heterostructures shows a higher polarization at room temperature, which makes them even more practical for device applications.

### Near-unity polarization from PbI_2_/WSe_2_ heterostructures

**Fig. 5:**
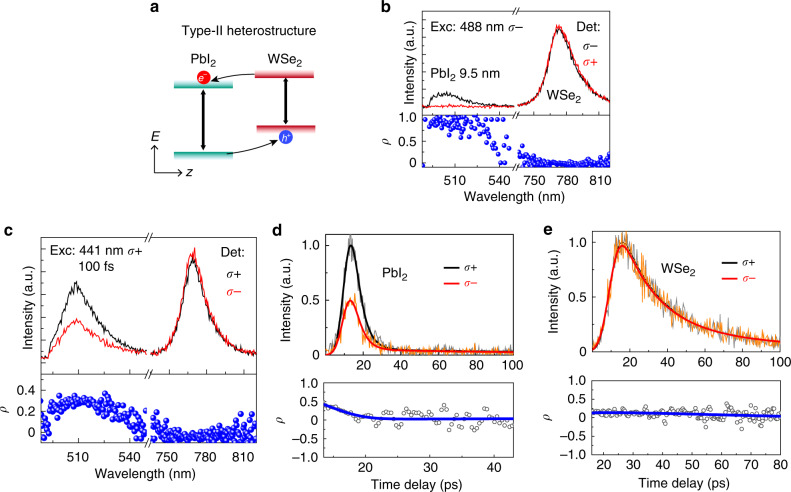
Degree of spin polarization and the dynamics in PbI_2_/WSe_2_ heterostructures. **a** Schematic of the type-II band alignment and the corresponding photogenerated carrier behaviors in PbI_2_/WSe_2_ heterostructures. **b** Circularly polarized PL spectra from a 9.5 nm thick PbI_2_/WSe_2_ heterostructure, and the corresponding calculated *ρ* as a function of wavelength. The excitation is 488 nm *σ*− polarized CW laser. **c**–**e** Circularly polarized PL spectra (**c**) and TRPL data from PbI_2_ (**d**) and WSe_2_ (**e**) in PbI_2_/WSe_2_ heterostructure, and the corresponding degree of polarization *ρ* calculated from PL and TRPL spectra. Excitation with 441 nm *σ*+ polarization femtosecond laser pulses.

We have studied the degree of polarization in the PL spectra emitted from PbI_2_/WSe_2_ heterostructures, which have the type-II band alignment that photogenerated electrons can transfer from WSe_2_ to PbI_2_ and holes transfer from PbI_2_ to WSe_2_ (Fig. 5a). The formation of type-II alignment also reduces the carrier lifetime in PbI_2_, due to the extra decay channel^38^. Therefore, a high degree of polarization is expected for PbI_2_ PL in PbI_2_/WSe_2_. Figure 5b shows the circular polarization-resolved PL emission of a 9.5-nm-thick heterostructure, under σ− polarized continuous-wave (CW) laser excitation at 488 nm. For PbI_2_ PL emission, we observe that σ− polarization (black) dominates the whole emission. The calculated degree of polarization (Fig. 5b, lower panel) approaches 100% at room temperature, which is due to the largely reduced carrier lifetime with a very small *τ*_c_/*τ*_s_. Similar to PbI_2_/WS_2_ heterostructures, the thicker heterostructures show a slightly smaller polarization value (Supplementary Fig. 12). The valley polarization of WSe_2_ is very low because the excitation light is far from resonance, even though its carrier lifetime reduces as well. We further performed time-resolved σ+ and σ− polarized PL emission experiments under the excitation of σ+ polarized 100-fs laser at 441 nm. The integrated PL intensity shows a degree of polarization of about 0.3 for PbI_2_, and almost zero degree of polarization for WSe_2_ (Fig. 5c). After the deconvolution of the measured decay curve from the IRF and the fitting (Fig. 5d), we obtain the σ+ and σ− polarized PL emission with 7.5 and 9.44 ps lifetime, and the polarization lifetime of 13 ps in the PbI_2_/WSe_2_ heterostructure. Considering the smaller value of *P*_0_ under non-resonance excitation, the obtained *τ*_c_ and *τ*_s_ is general in agreement with the observed degree of polarization. For WSe_2,_ the σ+ and σ− polarized PL emissions show similar decay curves of about 30 ps (Fig. 5e), showing a non-observable polarization lifetime, which agrees with the integrated PL spectra and the close to zero degree of polarization. With temperature-dependent polarization measurements, we find that the *ρ* of PbI_2_ PL decreases with the temperature decreases (Supplementary Fig. 13), which is consistent with the temperature dependence in PbI_2_/WS_2_ heterostructure.

## Discussion

In conclusion, we have demonstrated the realization of a near-unity spin polarization in layered PbI_2_/WS_2_ and PbI_2_/WSe_2_ in vdW heterostructures at room temperature via vdW engineering. The underlying physics relies on the reduction of the carrier lifetime through interlayer carrier transfer in vdW heterostructures. We observe a thickness-dependent degree of polarization of PbI_2_ in PbI_2_/WS_2_ heterostructures, that the thicker the PbI_2_ layers the smaller the degree of polarization. By time-resolved circulary polarization measurements, we reveal the spin polarization lifetime of PbI_2_ in heterostructures. The degree of polarization is also found to have an excitation power and wavelength dependence. The higher the excitation power leads to a slight decrease in the polarization. For excitations with photon energy away from the resonance, we observed a smaller polarization. The polarization decreases to zero under the excitation of 400 nm laser. Furthermore, we also demonstrated that the near-unity degree of polarization can be obtained in a PbI_2_ and monolayer-WSe_2_ heterostructure with a type-II band alignment, which can also be understood by manipulating the carrier lifetime.

The realization of high-spin polarization via the formation of heterostructure in principle can be applied with other substrates that quenches the PL. However, one needs to consider the balance between the increasing degree of polarization and the PL quenching. For PbI_2_/TMDCs heterostructures, the PL in PbI_2_ is quenched about one order of magnitude but the remaining intensity is still comparable with that of typical monolayer TMDCs. On the other hand, monolayer TMDCs provide an excellent platform to construct a high-quality heterostructure with PbI_2_ through vdW interaction, facilitates the efficient transport of carriers.

Moreover, for future applications, not only the high-spin polarization, but also spin coherence are of great importance. The detailed study of the spin coherence should take into account the effect of the temperature-related dephasing and is an interesting topic for future study. Our study provides a promising strategy via vdW engineering to achieve a high degree of polarization in vdW 2D materials at room temperature, which could provide valuable information for spintronics.

## Methods

### Sample preparation

The PbI_2_/TMDCs heterostructures were synthesized by a two-step PVD method. Briefly, monolayer TMDCs (WS_2_, WSe_2_) serving as the bottom layers for the heterostructures were synthesized first by PVD method. In the second step, lead iodide powder was placed in the central area of the tube furnace, the substrates with grown monolayer TMDCs from the first step were placed in the downstream area of the tube furnace. The pressure in the furnace was drawn to 40 mtorr by a vacuum pump and the argon flow rate was maintained at 20 sccm. The heating lasts 10 min at a temperature of 400 °C. By varying the deposition temperature of PbI_2_ from 180 to 220 °C, we obtained a growth trend of thin to thick PbI_2_/TMDCs heterostructures. The thicknesses of the heterostructures were measured with an atomic force microscope (AFM). By the same experimental method, pure PbI_2_ layers can be grown on SiO_2_/Si or mica substrates. In order to improve the environmental stability of the prepared PbI_2_/WS_2_ and PbI_2_/WSe_2_ heterostructures in optical measurement, all samples were encapsulated with polydimethylsiloxane (PDMS).

### Steady-state circular polarization-resolved PL spectroscopy

Steady-state circular polarization-resolved PL measurements were conducted using a confocal microscope (WITec, alpha-300) equipped with a ×50 objective (Zeiss, 0.75 NA). To create the circularly polarized excitation light source, the laser beam first passes through a linear polarizer (GTH10M-AM-A, Thorlabs), and then a quarter-wave plate (AQWP05M-600, Thorlabs). The circularly polarized beam is reflected by a cube beam splitter (CCM1-BS013/M, Thorlabs) and focused on the sample. The signal is collected by the same objective and is detected by a spectrometer (UHTS300) after passing through a razor edge long pass 488 nm laser filter, a quarter-wave plate and a polarizer (WP25M-UB, Thorlabs). Left-handed and right-handed PL components of the signal are distinguished by rotating the polarizer. Continuous-wave Ar ion laser (Coherent, Sapphire LP) at 488 nm (excitation power of 2 μW) is normally used as the excitation source. For comparison, 400 nm and 441 nm fs lasers pulses (excitation power of about 20 μW) were used as well. During the experiment, 150 grooves/mm gratings were used for PL measurements.

### Low temperature circular polarization-resolved PL measurement

The circularly polarized light is focused by a long working distance objective lens (×50, Zeiss, NA 0.55) onto the sample located in the cryostat (ST-500, Janis Research Company). The method of circular polarization-resolved PL measurement is the same as that at room temperature.

### Dynamics of circular polarization-resolved PL

The dynamics of circular polarization-resolved PL of heterostructures were measured using the same optical microscope (WITec, alpha-300) equipped with a streak camera (C10910, Hamamatsu). A mode-locked Ti: sapphire laser (Tsunami 3941-X1BB, Spectral Physics) (pulse width 100 fs, repetition rate 80 MHz) was used as the fundamental excitation source. Photons at 400 and 441 nm were generated via the frequency doubling from the fundamental 800 and 882 nm with a BBO crystal. The same optical paths were used to generate circularly polarized 400 and 441 nm excitation beams. The left- and right-handed circular polarization PL were guided into the streak camera for time-resolved measurements, which were distinguished by the same optical system described for the steady-state measurements.

## Supplementary information

Supplementary Information

## Data Availability

The data that support the findings of this study are available from the corresponding author upon reasonable request.
