# Supplementary material for: Room temperature near unity spin polarization in 2D Van der Waals heterostructures
Source: Nat Commun. 2020 Sep 7;11:4442. doi: 10.1038/s41467-020-18307-w (PMC7477097; doi:10.1038/s41467-020-18307-w)
Supplement: Supplementary file 1 — Supplementary Information [file 41467_2020_18307_MOESM1_ESM.pdf]

Supplementary Information

Room Temperature Near Unity Spin Polarization in 2D Van der Waals Heterostructures

Danliang Zhang[1], Ying Liu[2], Mai He[1], Ao Zhang[3], Shula Chen[2], Qingjun Tong[1], Lanyu Huang[1], Zhiyuan Zhou[1], Weihao Zheng[2], Mingxing Chen[3], Kai Braun[4], Alfred J. Meixner[4], Xiao Wang[1], Anlian Pan[2]**

[1]Key Laboratory for Micro-Nano Physics and Technology of Hunan Province, School of Physics and Electronics, Hunan University, Changsha 410082, China

[2]Key Laboratory for Micro-Nano Physics and Technology of Hunan Province, College of Materials Science and Engineering, Hunan University, Changsha 410082, China

[3]Key Laboratory for Matter Microstructure and Function of Hunan Province, School of Physics and Electronics, Hunan Normal University, Changsha 410081, China

[4]Institute of Physical and Theoretical Chemistry and LISA+, University of Tübingen, Auf der Morgenstelle 18, 72076 Tübingen, Germany

*Email: xiao_wang@hnu.edu.cn; anlian.pan@hnu.edu.cn

Table of Contents

Supplementary Note 1. DFT calculations of PbI_2

Supplementary Note 2. Numerical calculations for carrier diffusion and the corresponding degree of polarization

Supplementary Note 3. Schematic of the experimental set-up

Supplementary Note 4. Optical characteristics of pure PbI_2

Supplementary Note 5. Optical characteristics of pure WS_2 atomic layers

Supplementary Note 6. Thickness dependent TRPL spectra of pure PbI_2

Supplementary Note 7. Excitation power dependent degree of polarization

Supplementary Note 8. Excitation wavelength dependent degree of polarization

Supplementary Note 9. Polarization dynamics of pure PbI_2

Supplementary Note 10. Thickness dependent polarization of PbI_2 in PbI_2/WSe_2 heterostructures

Supplementary Note 11. Temperature dependent circularly polarized PL spectra of PbI_2/WSe_2 heterostructures.

Supplementary Note 1. DFT calculations of PbI_2

Density-functional theory (DFT) calculations of PbI_2 were performed using Vienna ab initio Simulation Package (VASP)[1, 2]. A slab was used to model the thin films of PbI_2. The exchange correlation functional is approximated by the generalized gradient approximation as parametrized by Perdew, Burke and Ernzerhof[3], and pseudopotentials were constructed by the projector augmented wave method[4, 5]. The Brillouin zone is sampled by a mesh of 12×12×8 for the bulk and a mesh of 12×12×1 for the thin films.

The band structure of the PbI_2 thin film with a thickness of 20 layers is shown in the main manuscript. We have also performed calculations for the bulk phase and the 4L-PbI_2. Supplementary figure 1 shows the geometric structures and the supplementary figure 2 shows the calculated band structures. The geometric structure Spin-orbit coupling (SOC) was included in our calculations. Our results indicate that both are direct gap semiconductors. For the bulk phase, the valance band minimum (VBM) and the conduction band minimum (CBM) are located at the A point. Our results are in good agreement with previous studies[6, 7]. Supplementary figure 2 (c-f) show the orbital projected band structure for the two phases. Essentially, it reveals that for the bulk phase the VBM at A is mainly composed of I-*P*z orbitals, while the CBM is mainly contributed by p orbitals of Pb atoms. The situation is slightly different at Γ point from that at the A point in that the VBM is mainly contributed by the I-*P*z orbital.

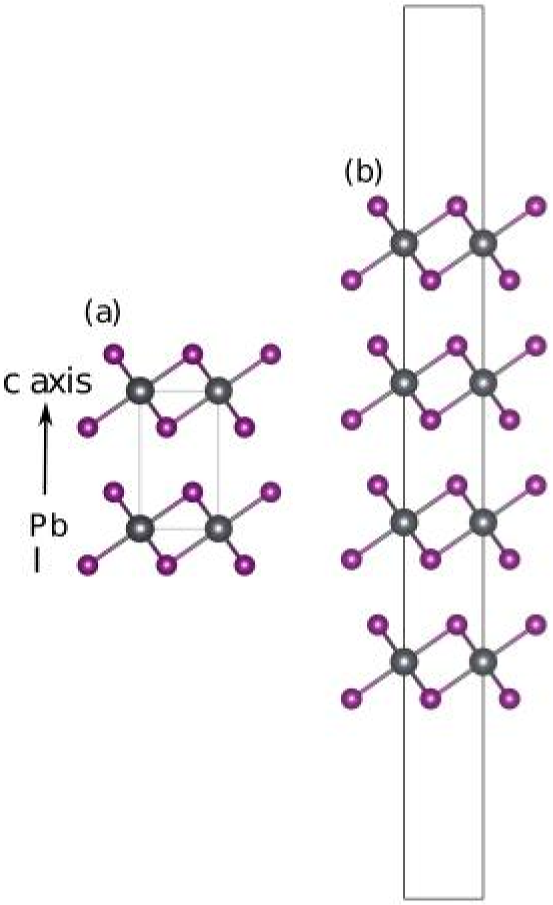

Supplementary figure 1. The geometric structure for (a) the bulk PbI_2 and (b) the 4L-PbI_2. The lattice constants are a = b = 4.56 Å for both the bulk phase and the thin film. The lattice constant along the c axis is 6.98 Å for the bulk phase. The thickness of the 4L-PbI_2 is about 24.63 Å, which together along with a vacuum region of 20 Å gives rise to 44.63 Å for the lattice constant along the c axis in our DFT calculations.

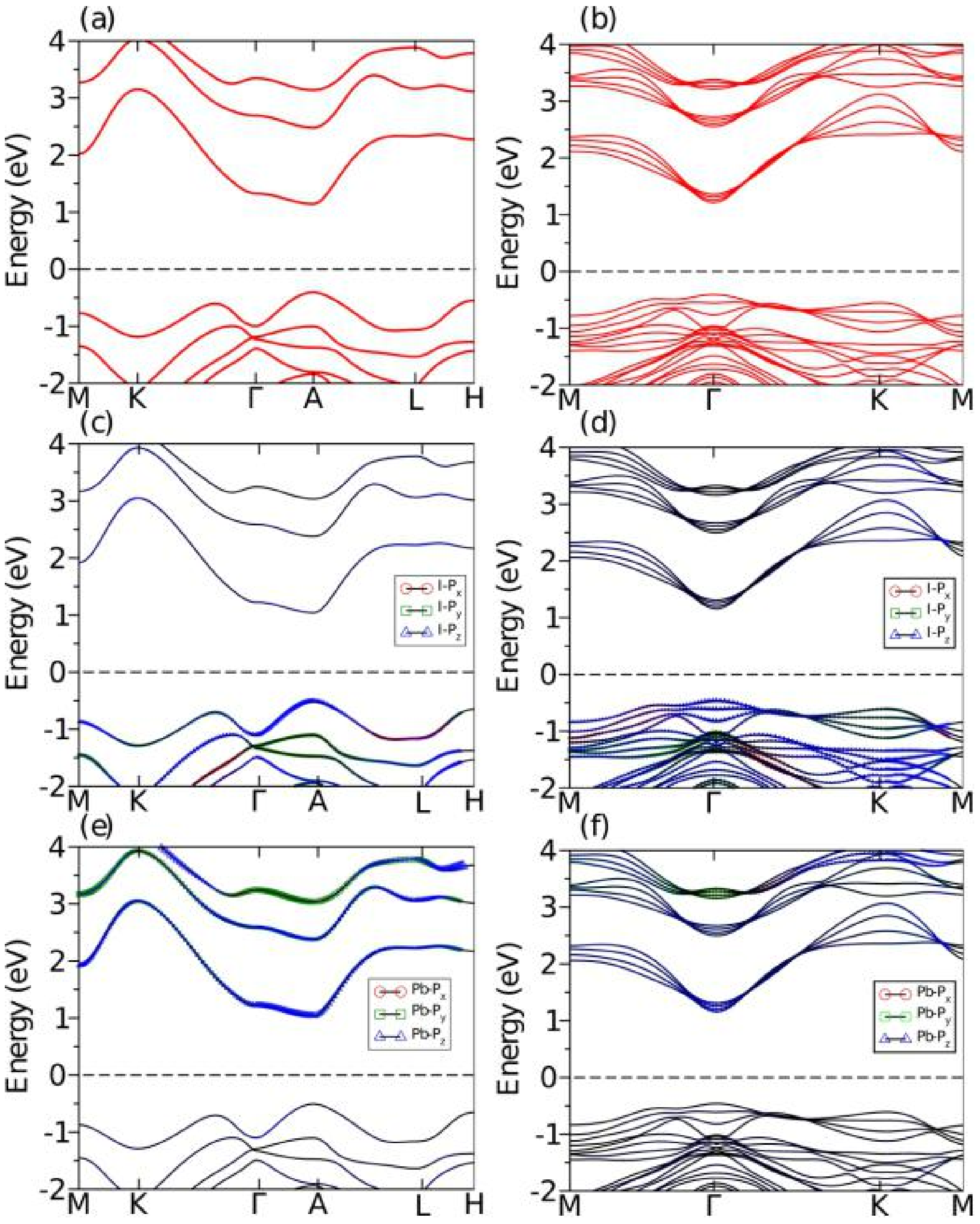

Supplementary figure 2. Band structure for (a) the bulk phase and (b) the 4L-PbI_2. (c)-(f) show the orbital-projected band structure for the two phases.

Supplementary Note 2. Numerical calculations for carrier diffusion and the corresponding degree of polarization

In order to quantitatively analyze the circularly polarized PL of PbI_2 layers in PbI_2/WS_2 heterostructure with different thicknesses, we built a numerical calculation model based on the population and distribution of carriers in PbI_2 layers, which can be described by a one-dimensional diffusion supplementary equation as following:

$$\frac{\partial n(x,t)}{\partial t} = D\frac{\partial^2 n(x,t)}{\partial x^2} - \eta n^2(x,t) \tag{1}$$

where $n(x,t)$ is the carrier population at the depth of x nm of the PbI_2 layers with time delay t. The initial carrier distribution of the PbI_2 layers is given by $n(x,0) = n_0 \exp(-\alpha x)$ with absorption coefficient α being 4.09×10^5 cm^{-1}. The average value of the carrier diffusion coefficient D of PbI_2 is 0.039 cm^2s^{-1}. The values of α and D are taken from the literature[8]. The bimolecular recombination coefficient η is obtained as 2.55×10^{-1} cm^2s^{-1} by fitting the decay curves of PbI_2 in PbI_2/WS_2 heterostructure with thickness larger than 200 nm in which the carrier injection to WS_2 can be neglected[8]. The carriers close to the bottom layer of PbI_2 were assumed to transfer to WS_2 layer instantly via type-I charge transfer, therefore the boundary condition is given as $n(X - X_{\min}, t) = 0$. Here X is the thickness of the PbI_2 layers, and $X_{\min}$ = 9 nm is the minimum thickness of the PbI_2 layers, above which the time-resolved photoluminescence (TRPL) measurements can be conducted under laboratory conditions. τ_{eff} denotes the effective lifetime of the carriers in the PbI_2 layers, which is weighted averagely based on $n(x,t)$ as

$$\tau_{eff}(X) = \frac{1}{N_0}\int_{t=0}^{t=t_d}\int_{x=0}^{x=X-X_{\min}} n(x,t)\,dx\,dt \tag{2}$$

where N_0 is the normalization constant. The PL circularly polarized degree P_{PL} can be described in its simplest form by the supplementary equation[9]

$$P(X) = \frac{P_0}{1+\tau_{eff}(X)/\tau_s} \tag{3}$$

In our case $P_0 = 100\%$, τ_s is the carrier spin relaxation time. By solving supplementary equation 1, and substituting the result to supplementary equation 2 and

3, the circularly polarized PL of PbI_2/WS_2 heterostructure with different thicknesses is shown in Figure 2(c) in the main paper. The experiment data is also depicted in Figure 2(c) for comparison. Here time t_d=10 ps, τ_s=15 ps.

Supplementary Note 3. Schematic of the experimental set-up

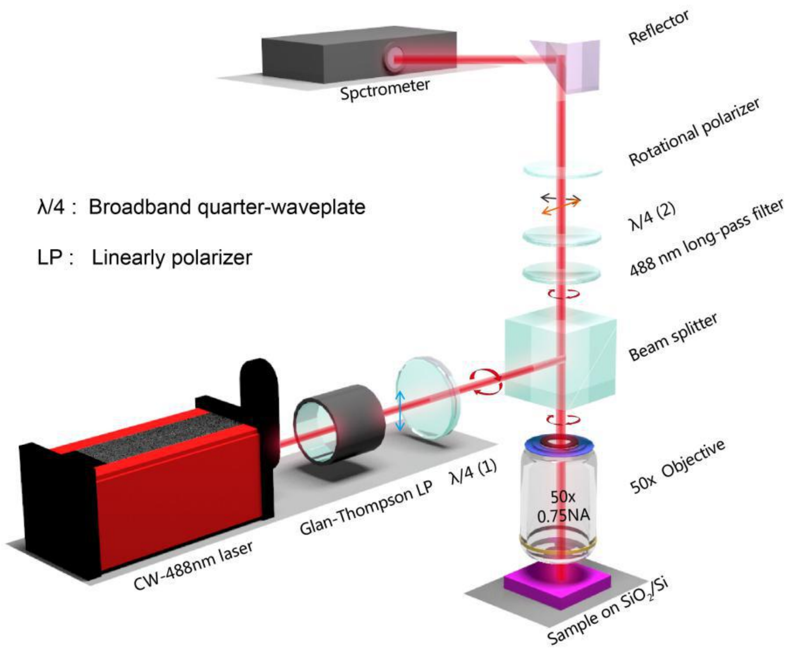

Supplementary figure 3. Experimental setup for helicity-resolved micro-PL spectroscopy.

Supplementary Note 4. Optical characteristics of pure PbI_2

We synthesized pure PbI_2 samples on SiO_2/Si and mica substrates using the PVD method for comparison. Supplementary figure 4 (a) and (c) show the optical images of the grown PbI_2 on SiO_2/Si substrate and mica, respectively. Supplementary figure 4 (b), (d) show the corresponding atomic force microscopy (AFM) images, indicating PbI_2 thickness of 230 nm and 44.7 nm on SiO_2/Si substrate and mica substrate, respectively. Micro Raman spectroscopy was performed for further characterization of PbI_2 samples on different substrates (Supplementary figure 4 (e)). Three feature peaks associated with PbI_2 are identified: E_g peak located at 75 cm^{-1}, A_{1g} peak located at 95 cm^{-1}, and E_u + A_{2u} peak located at 105 cm^{-1}. The observed peaks are consistent with those reported in the previous work[10], and show no obvious changes on the SiO_2/Si and mica substrate.

In order to compare the effects of different substrates on the spin polarization of PbI_2, we measured the circularly polarized PL spectra of PbI_2 on the SiO_2/Si and mica substrate. We measured the circularly polarized PL spectra of PbI_2 under σ- polarized light laser excitation at 488 nm. The results are shown in supplementary figure 5 (a) and (b). The degree of polarization ρ of pure PbI_2 is only about 10%, and there is no significant difference between SiO_2/Si and mica substrates. In addition, we investigated the effect of different wavelengths of excitation light on the spin polarization of PbI_2 (Supplementary figure 5 (c, d)). The ρ of PbI_2 on different substrates is close to zero under continuous wave (CW) laser excitation at 405 nm due to the off-resonant excitation.

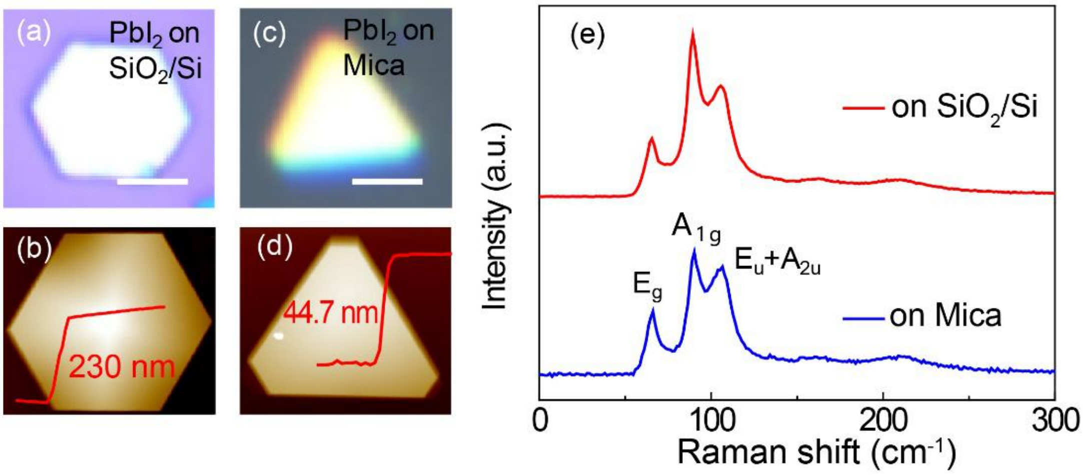

Supplementary figure 4. (a), (c) Optical images of PbI_2 on SiO_2/Si and Mica substrates, respectively. All scale bar:10 μm. (b), (d) Corresponding AFM images of (a) and (c). (e) Raman spectra of PbI_2 on SiO_2/Si and Mica substrates, respectively. The excitation is a 532 nm CW laser.

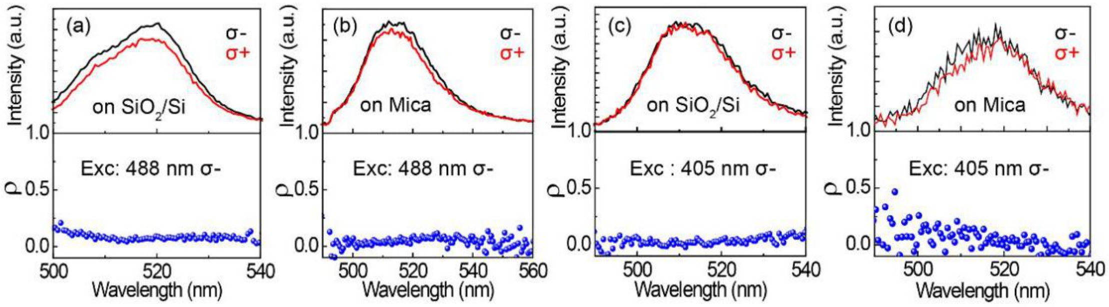

Supplementary figure 5. Room-temperature circularly polarized PL spectra from PbI_2 on SiO_2/Si (a), (c) and Mica substrates (b), (d) under the excitation of 488 nm and 405 nm σ- polarization CW laser. For each situation, the degree of polarization ρ calculated from the corresponding polarization-resolved spectra is shown in the lower panel.

Supplementary Note 5. Optical characteristics of pure WS_2 atomic layers

For comparison, we synthesized WS_2 atomic layers on Si wafers with different thicknesses by changing the deposition temperature. We measured the circular polarization resolved PL spectra of the monolayer and bilayer WS_2 excited by σ-polarized light at room temperature. For the as-grown monolayer WS_2, the calculated ρ of the A-exciton PL emission is about 15% (Supplementary figure 6 (a)). For bilayer WS_2, we observe three PL emission peaks assigned to the B-exciton, A-exciton, and indirect band transition and the calculated degree of polarization ρ are about 40%, 12%, and 0%, respectively (Supplementary figure 6 (b)). These observations are consistent with the previous studies[11,12].

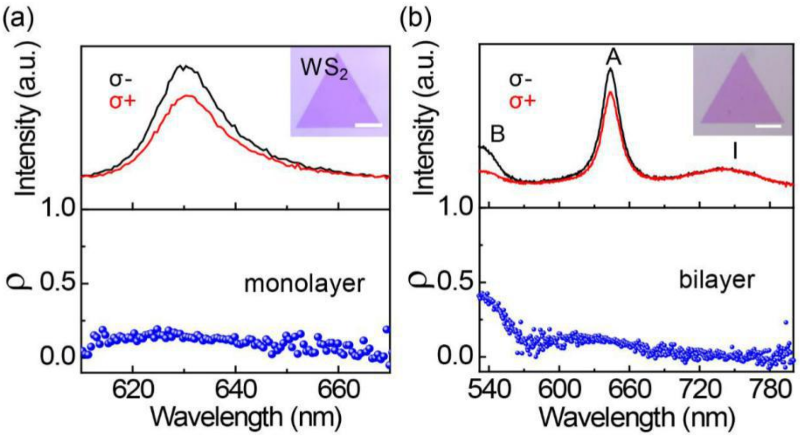

Supplementary figure 6. Circularly polarized PL spectra from monolayer (a) and bilayer (b) WS_2, and the corresponding degree of polarization ρ calculated from PL spectra from WS_2. The insets are the optical images. All scale bar: 10 µm. The excitation is 488 nm σ- polarization CW laser at room temperature.

Supplementary Note 6. Thickness dependent TRPL spectra of pure PbI_2

We measured the time-dependent spectra of pure PbI_2 with different thicknesses. Supplementary figure 7 shows the normalized TRPL of PbI_2 with typical thicknesses of 13.2 nm, 31.5 nm and the bulk. We found similar TRPL curves with a fast decay time of about 45 ps, indicating that the intrinsic recombination process of PbI_2 is basically the same within the range of thickness in this study.

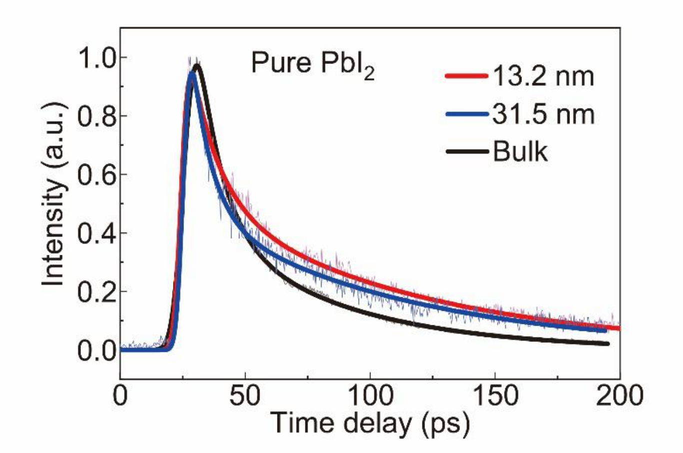

Supplementary figure 7. TRPL data from pure PbI_2 with different thicknesses at room temperature. The excitation light source is 440 nm femtosecond laser.

Supplementary Note 7. Excitation power dependent degree of polarization

We measured the degree of polarization of PbI_2 in PbI_2/WS_2 heterostructures at different excitation powers. Increasing the power of the σ- polarized excitation from 2 μW to 10 μW, we observe the initial very weak σ+ polarized PL emission increases (Supplementary figure 8), indicating a decreasing degree of polarization at higher excitation power. Supplementary figure 8 (b) shows the calculated degree of polarization from sequence excitation powers. The slightly reduced degree of polarization from 0.999 to 0.913 can be understood by the decreased spin polarization lifetime due to a higher chance of carrier spin flip with a higher density of carrier at high excitation powers. Similar results have been observed in different systems, such as GaAs nanostructures[13].

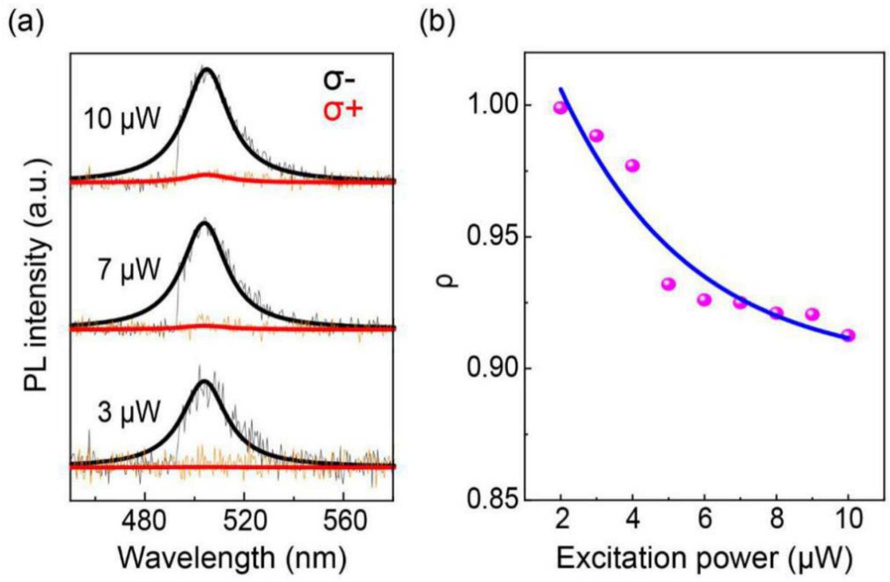

Supplementary figure 8. (a) Typical power-dependent circularly polarized PL spectra from PbI_2 in PbI_2/WS_2 heterostructures at room temperature. (b) Degree of polarization ρ of PbI_2 as a function of excitation power.

Supplementary Note 8. Excitation wavelength dependent degree of polarization

We measured the circular polarization of the heterostructures under 400 nm femtosecond pulsed laser excitation which is further away from the resonance excitation conditions compared with the 441 nm excitation shown in the main text. Supplementary figure 9 shows the circularly polarized PbI_2 PL spectra and their dynamics in layered PbI_2/monolayer-WS_2 (a, b) and PbI_2/monolayer-WSe_2 (c, d) heterostructures. For both cases, we observed that the σ- and σ+ polarized PL signals were similar, showing no observable polarization. The time-resolved σ- and σ+ polarized PL spectra of PbI_2 from the heterostructures shows about 22 ps (supplementary figure 9 (b)) and 26 ps (Supplementary figure 9 (d)) in PbI_2/monolayer-WS_2 and PbI_2/monolayer-WSe_2 heterostructures, respectively.

We summarized the ρ of layered PbI_2/monolayer-WS_2 heterostructures at different excitation wavelengths in supplementary figure 10, showing ρ being about 0.998, 0.358, and 0.091 under the excitation of 488 nm, 441 nm, and 400 nm lasers, respectively.

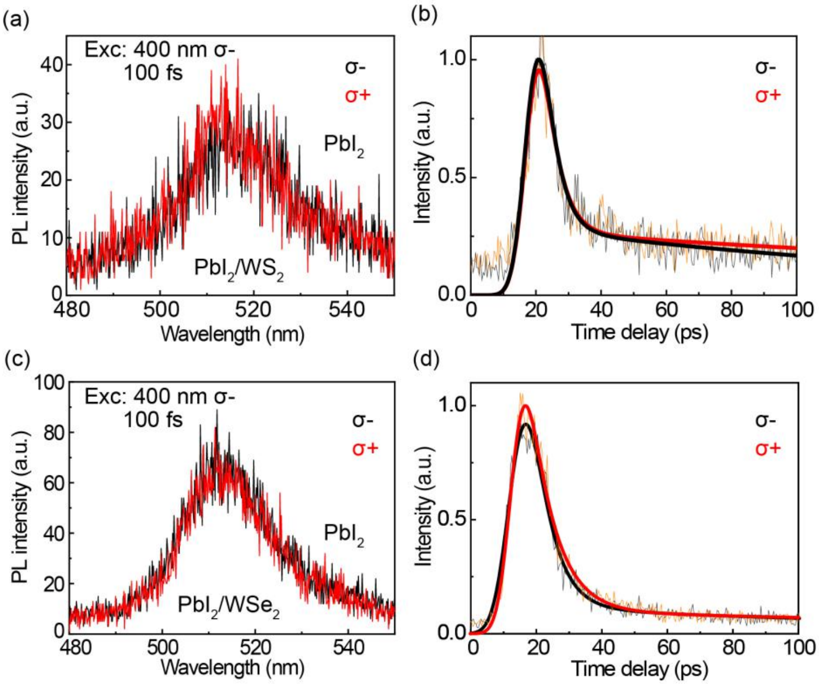

Supplementary figure 9. Room-temperature circularly polarized PL spectra (a, c) and the corresponding TRPL data (b, d) from PbI_2/WS_2 and PbI_2/WSe_2 heterostructures. The PL spectra taken from the streak camera image. The excitation light source is 400 nm $\sigma-$ polarization femtosecond laser with a power of 24 μW.

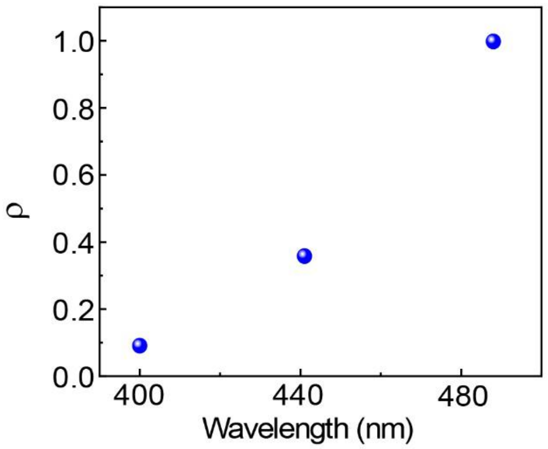

Supplementary figure 10. Degree of polarization ρ of PbI_2 in PbI_2/WS_2 heterostructure as a function of excitation wavelength.

Supplementary Note 9. Polarization dynamics of pure PbI_2

We measured the temperature-dependent and time-resolved circularly polarized PL of pure PbI_2 samples under the excitation of σ- polarized 100-fs laser at 440 nm. Supplementary figure 11 shows the temperature-dependent circularly polarized TRPL (a-d)) from pure PbI_2 at typical temperatures and the corresponding degree of polarization ρ (e-f)) as a function of time.

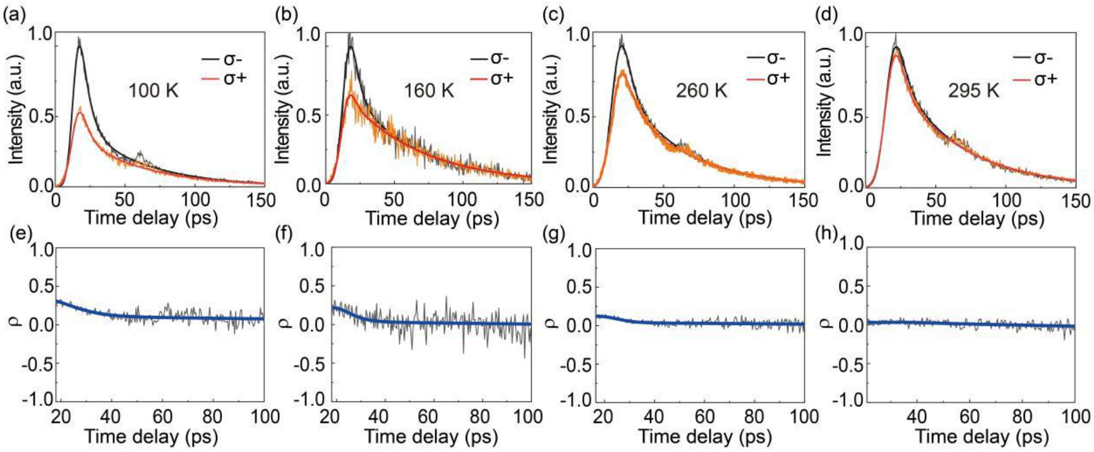

Supplementary figure 11. (a)-(d) Circularly polarized TRPL data from pure PbI_2 at typical temperatures. (e)-(f) Corresponding degree of polarization ρ as a function of time calculated from the TRPL spectra. Excitation: 440 nm σ- polarization femtosecond laser pulses.

Supplementary Note 10. Thickness dependent polarization of PbI_2 in PbI_2/WSe_2 heterostructures.

We prepared PbI_2/WSe_2 heterostructures with different thicknesses for circular polarization measurement. The ρ of PbI_2 decreased with increasing thickness due to the increase of carrier lifetime, which is similar to PbI_2/WS_2 heterostructures. Supplementary figure 12 shows the calculated PbI_2 the ρ in heterostructures with different thicknesses. When the thickness of PbI_2 in the heterostructure increases from 9.5 nm to 30.9 nm, the ρ decreases from 0.998 to 0.758.

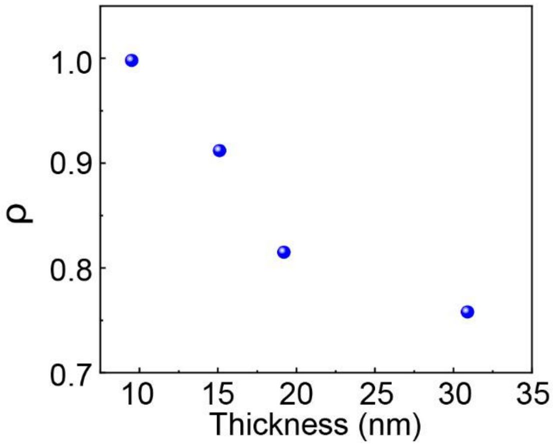

Supplementary figure 12. Degree of polarization ρ of PbI_2 as a function of thickness of the PbI_2/WSe_2 heterostructures. Excitation: 488 nm σ- polarization CW laser at room temperature.

Supplementary Note 11. Temperature dependent circularly polarized PL spectra of PbI_2/WSe_2 heterostructures.

Supplementary figure 13 shows the circularly polarized PL spectrum from a PbI_2/WSe_2 heterostructure at typical temperatures. We find that the ρ of PbI_2 PL in heterostructures decreases from 83.2% to 66.6% when the temperature decreases from 227 K to 77 K. As temperature decrease, interlayer charge transfer decreases, and the polarization decreases accordingly.

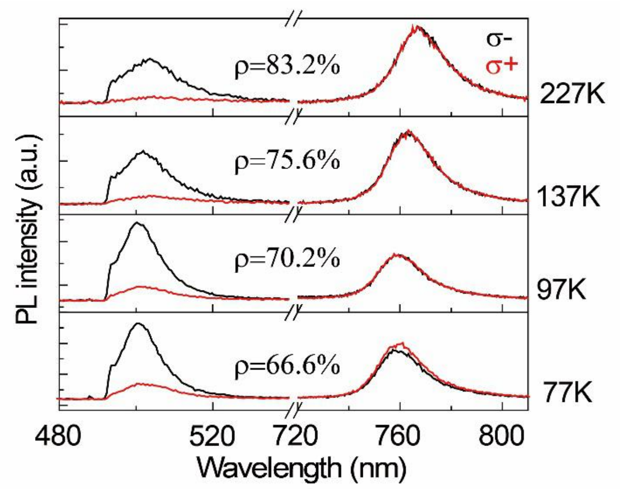

Supplementary figure 13. Circularly polarized PL spectra of PbI_2/WSe_2 heterostructure at different temperatures under the excitation of a 488 nm σ- polarized CW laser beam.

Supplementary Reference

1. G.Kresse JF. Efficiency of ab-initio total energy calculations for metals and semiconductors using a plane-wave basis set. *Computational Materials Science* **6**, 15-50 (1996).

2. G. Kresse JF. Efficient iterative schemes for ab initio total-energy calculations using a plane-wave basis set. *Phys Rev B* **54**, 11169-11186 (1996).

3. John P. Perdew KB, Matthias Ernzerhof. Generalized Gradient Approximation Made Simple. *Phys Rev Lett* **77**, 3865-3868 (1996).

4. G. Kresse DJ. From ultrasoft pseudopotentials to the projector augmented-wave method. *Phys Rev B* **59**, 1758-1775 (1999).

5. Blochl PE. Projector augmented-wave method. *Phys Rev B Condens Matter* **50**, 17953-17979 (1994).

6. Yagmurcukardes M, Peeters FM, Sahin H. Electronic and vibrational properties of PbI_2: From bulk to monolayer. *Phys Rev B* **98**, 085431 (2018).

7. Zhou M, Duan W, Chen Y, Du A. Single layer lead iodide: computational exploration of structural, electronic and optical properties, strain induced band modulation and the role of spin-orbital-coupling. *Nanoscale* **7**, 15168-15174 (2015).

8. Zheng, W. et al. Probing and Manipulating Carrier Interlayer Diffusion in van der Waals Multilayer by Constructing Type-I Heterostructure. *Nano Lett.* **10**, 7217-7225 (2019).

9. F. Meier, B. P. Zakharchenya. Optical orientation (Elsevier, North-Holland, 1984).

10. Xiao H, Liang T, Xu M. Growth of Ultraflat PbI_2 Nanoflakes by Solvent Evaporation Suppression for High-Performance UV Photodetectors. *Small* **15**, e1901767 (2019).

11. Zhu B, Zeng H, Dai J, Gong Z, Cui X. Anomalously robust valley polarization and valley coherence in bilayer WS_2. *Proc Natl Acad Sci* **111**, 11606-11611 (2014).

12. Mak KF, He K, Shan J, Heinz TF. Control of valley polarization in monolayer MoS_2 by optical helicity. *Nat Nanotechnol* **7**, 494-498 (2012).

13. Chen SL, Kiba T, Yang XJ, Takayama J, Murayama A. Power-dependent spin

amplification in (In, Ga)As/GaAs quantum well via Pauli blocking by tunnel-coupled quantum dot ensembles. *Appl Phys Lett* **108**, 152103 (2016).
